# Designing Clinically Valuable Telehealth Resources: Processes to Develop a Community-Based Palliative Care Prototype

**DOI:** 10.2196/resprot.3266

**Published:** 2014-09-04

**Authors:** Jennifer Joy Tieman, Deidre Diane Morgan, Kate Swetenham, Timothy Hong Man To, David Christopher Currow

**Affiliations:** ^1^Discipline of Palliative and Supportive ServicesDepartment of Health SciencesFlinders University of South AustraliaAdelaideAustralia; ^2^Southern Adelaide Palliative ServicesAdelaideAustralia

**Keywords:** telemedicine, palliative care, delivery of care, home care

## Abstract

**Background:**

Changing population demography and patterns of disease are increasing demands on the health system. Telehealth is seen as providing a mechanism to support community-based care, thus reducing pressure on hospital services and supporting consumer preferences for care in the home.

**Objective:**

This study examined the processes involved in developing a prototype telehealth intervention to support palliative care patients involved with a palliative care service living in the community.

**Methods:**

The challenges and considerations in developing the palliative care telehealth prototype were reviewed against the Center for eHealth Research (CeHRes) framework, a telehealth development model. The project activities to develop the prototype were specifically mapped against the model’s first four phases: multidisciplinary project management, contextual inquiry, value specification, and design. This project has been developed as part of the Telehealth in the Home: Aged and Palliative Care in South Australia initiative.

**Results:**

Significant issues were identified and subsequently addressed during concept and prototype development. The CeHRes approach highlighted the implicit diversity in views and opinions among participants and stakeholders and enabled issues to be considered, resolved, and incorporated during design through continuous engagement.

**Conclusions:**

The CeHRes model provided a mechanism that facilitated “better” solutions in the development of the palliative care prototype by addressing the inherent but potentially unrecognized differences in values and beliefs of participants. This collaboration enabled greater interaction and exchange among participants resulting in a more useful and clinically valuable telehealth prototype.

## Introduction

Given an aging population and changing patterns of disease, health systems are being challenged by an increasing number and type of care needs. Part of the policy response has been to try to stabilize the demand on hospitals by building capacity in primary care and by supporting care provision in the community. This in turn has driven the need for innovative approaches to facilitate care in community settings [1-3]. Telehealth is gaining increasing prominence within the health system as one solution, driven by the promise of benefits for patients, their families, health providers, and health services coupled with the possibility of cost savings [4-6]. The possibilities afforded by telehealth in community-based care have led to a rapid expansion of telehealth resources and options [7-11].

Community-based care includes care of patients with palliative care needs. Most patients in the final stage of their life will be cared for in their home for some or for all of this period. Many of these patients will have some form of engagement or interaction with a palliative care service. Involvement with a palliative care service has been shown to improve a person’s likelihood of dying at home and to reduce the symptom burden associated with advanced illness [12,13]. While care delivery for patients supported by palliative care services can be structured with a sequenced pattern of contacts or home visits, engagement is not continuous. Patients may decline rapidly or unexpectedly between scheduled visits with little opportunity for proactive intervention by the palliative care service. Patient self-reporting processes with real-time feedback would enable early identification of changes and facilitate targeted clinical and service responses, potentially enhancing care and outcomes. Such self-reporting could be provided through telehealth. Telehealth modules could allow patients to enter information about their symptoms and functional status with algorithms triggering automated clinical alerts based on the data entered. Furthermore, telehealth could potentially support family carers who are integral to enabling the care of palliative care patients at home [14,15]. Some clinical areas have already investigated the possibilities of telehealth-enabled patient self-report in the community [16-18]. These studies have shown a range of potential benefits including increased communication, early intervention, better symptom control, and enhanced patient satisfaction and empowerment. However, the telehealth evidence base within palliative care is more limited [19,20].

The processes involved in developing resources that are clinically meaningful and that interface with, or enhance, work practices are complex and multidimensional. Telehealth resources must satisfy the utility and usability criteria of clinicians and consumers of care as well as meet the policy and system requirements of funders. Telehealth modules may not be successfully deployed where there is a limited understanding of the physical and social structures of the clinical environment and a lack of appreciation of the implications of technical decisions on functional outcomes. Aligning clinical utility within technological capabilities requires consideration of many elements such as:

- the health context and current service delivery model
- what opportunities are enabled by changing practice and by incorporating technological capabilities
- whether knowledge and evidence exists that support both the clinical components and the telehealth choices
- the available technological frameworks and systems
- processes needed to ensure effective and timely decision making
- mechanisms for collaborative resolution of developmental issues
- support for iterative refinement of the telehealth resources before use in research studies and clinical practice.

Resources developed in isolation of the intended use and user, and simply released to the market to determine their potential use and value, may have limited value. However, those that are built to meet only a specific local clinical purpose may be too limited for sustainability and scalability. Westbrook and Braithwaite [21] have argued that there is a need to look at how information and communications technology can be conducted in real clinical settings that acknowledges the complex and collaborative work between colleagues and that involves clinicians from the frontlines in the developmental work. This means that concept selection, prototype design, and construction need to integrate clinical worth and technical feasibility.

A recent review and critical appraisal of eHealth frameworks with respect to the fit between human, organizational, and technological factors has highlighted the interdependent factors underpinning telehealth innovation [22]. The authors noted that while many studies in the review highlighted individual components such as collaboration between developers and researchers or input from users and stakeholders, these components were not reflected in cohesive approaches that collectively enhanced the likelihood of successful eHealth development. Consequently, based on their analysis of the studies, they defined a holistic framework to guide the development of eHealth technologies. Their framework, the Center for eHealth Research (CeHRes) roadmap, is an iterative model that maps the research and developmental activities involved in developing eHealth applications from concept definition through development to summative evaluation. These activities can be described as follows:

- Multidisciplinary project management: facilitates cooperation between those who build the technology and those who are using or affected by it.
- Contextual inquiry: entails gathering information from end users and building an understanding of the environment where the technology will be implemented.
- Value specification: identifies the underpinning value of the various stakeholders to define the critical purposes of the technology intervention.
- Design: assigning and testing the functional characteristics needed to develop a workable and usable prototype.
- Operationalization: deploying the prototype for use and supporting the implementation with training and education.
- Summative evaluation: assessing the effect and the impact of the technology in its environment.

This paper reports on the sequences involved in designing and developing the prototype of a telehealth intervention to support palliative care patients and their carers living in the community. It aims to identify the challenges and considerations in creating a palliative care telehealth prototype mapped against the four formative phases of the CeHRes roadmap, namely multidisciplinary project management, contextual inquiry, value specification, and design.

## Methods

**Figure 1:**
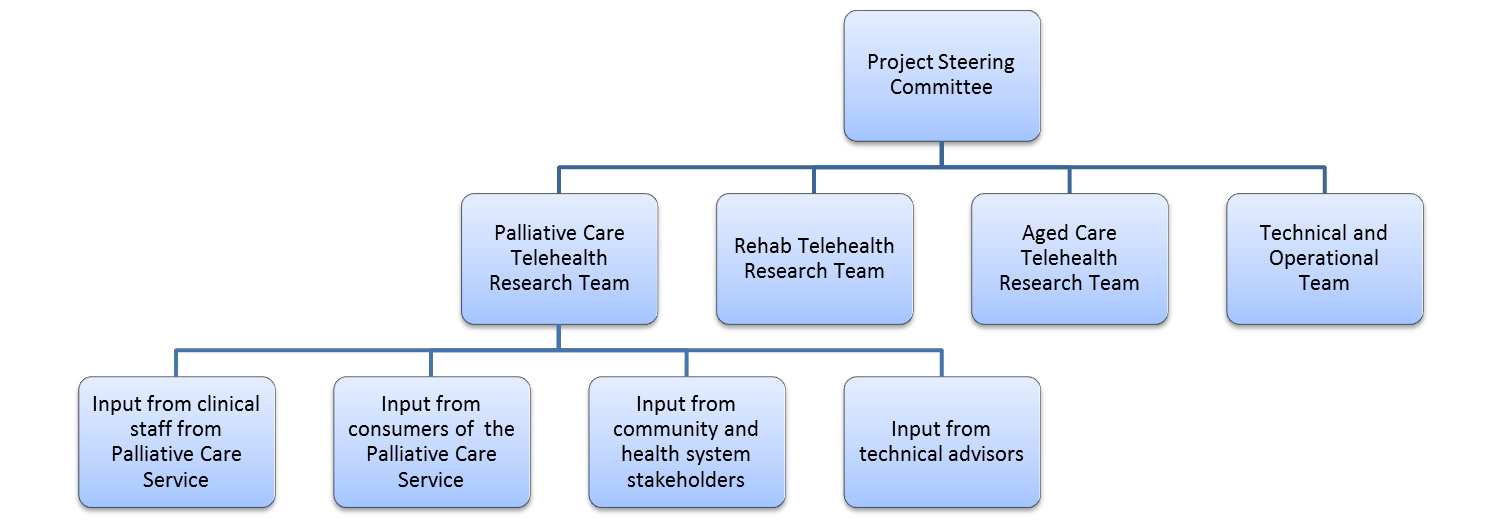
Overview of governance relationship and avenues for project input.

This palliative care telehealth module was developed as one part of the *Telehealth in the Home: Aged and Palliative Care in South Australia* project, which examined potential benefits associated with the integration, supplementation, or novel development of telehealth as a key component of care delivery to the home in three clinical care areas (ie, aged care, rehabilitation, and palliative care). Each work stream was led by a clinical research team supported by the project’s technical and operational team. Ethics approval was gained through Southern Adelaide Clinical Human Research Ethics committee, application number HREC/13/SAC/88(168.13). The whole project was overseen by the Project Steering Committee. Input and reporting relationships are outlined in Figure 1.

## Results

### Overview

Significant issues were identified in all aspects of the concept and prototype development that reflected the phases and activities outlined below against the CeHRes model elements. Examples of types of activities and decisions against these four stages for concept ideation and prototype development are included in Table 1.

**Table 1:** Examples of how inputs associated with research and development activities affected telehealth intervention.

	Concept development	Prototype development
Multidisciplinary project management	Recognizing that a finding from a randomized controlled trial (initiating a case conference at a point of functional decline identified by a standard tool) could be translated into an online app	Opportunities to test and evaluate different technical options for service against multiple criteria
	Staff from another eHealth project identified the importance of a Web-based system for data entry	Including a Quality Improvement/usability phase with patients in the community
Contextual inquiry	Awareness of palliative care service’s previous involvement in clinical studies	Clinicians should not be required to undertake atypical behavior patterns (eg, go to another building for a virtual service)
	Discussions with the clinical team identified that many had no experience with tablets and only limited computer experience in the workplace	Developing required specification of implicit clinical practice
	Health service providers were facing funding difficulties and hence were supportive of approaches to maintain or enhance community service provision	Assessing trade-offs between device functions and the capabilities of intended users
Value specification	Enhancing access to patient and carer’s state of health/well-being between visits	Ensuring continuity of care across patient and carer and pre/post-bereavement
	Supporting clinicians in moving to telehealth	Usability as the priority for prototype
	Doing more with less (or same)	“You’re not a geek, it’s ok to not know things”
Design	Using commonly available devices to support post-trial sustainability	Modifying features based on feedback from patients who assisted in a quality assurance phase
	Recognizing the usability requirements of older people who may have accessibility issues	Remote facility to update carer resources after death of a participant

### Multidisciplinary Project Management

The Palliative Care (Telehealth) Research Team (PCTRT) was established to guide the development of a telehealth model for use by the community team of a specialist palliative care service. Membership of the PCTRT included the Director of the Clinical Service, clinical staff (ie, medicine, nursing, allied health), and researchers with expertise in clinical trial design, health services research, and evaluation. A project manager was appointed to support the project development. Input was sought and received during concept and module development from service providers, stakeholders, and patients and carers involved with the service. Meetings were held with the clinicians providing direct care to enable input and feedback on the proposals and the development of the prototype.

The PCTRT had access to, and ongoing support from, members of the Technical and Operational Team who were responsible for the network architecture, systems, and applications used to deliver the telehealth interventions for each of the three clinical streams. The skill base for the PCTRT was enhanced by the inclusion of staff from an associated eHealth project providing access to additional resources and expertise [23].

### Contextual Inquiry

The contextual framework for the telehealth intervention was pivotal. Preliminary work on the environment and clinical needs had been undertaken in the process of grant application. Members of the PCTRT were integral to this process. This provided continuity from concept to prototype development and ensured that the original concept idea was rooted in clinical utility. The application process also meant that relationships among potential participants needed to be investigated and established and that key stakeholders needed to be contacted to formalize their support. This provided the prompt for meetings and workshops to explore aspects of telehealth in the local environment.

For those seeking to incorporate telehealth into clinical care practices, being able to use what has been shown to be effective from research in innovative telehealth solutions is critical. While technological innovation in itself may show potentials and opportunities, it is the quality of the clinical content of a telehealth module and its relevance to practice that ensures its value and contribution to the provider and patient community. Several elements of this palliative care module used findings from previous research studies in which the associated clinical service had been involved [24,25]. In effect, this meant that the module used research evidence that improved its clinical value. Further, incorporating the results of this research into the telehealth module offered a mechanism by which the research evidence could be translated for use in practice.

### Value Specification

While all stakeholders believed that incorporating telehealth in community service models offered the possibility of enhanced care, there were different views and attitudes on the shape, purpose, and outcomes of such interventions. The meetings and workshops held during the grant application development process facilitated the identification of values held by different participants. This was further explored in meetings held with service staff, technical teams, community members and providers, and funders and stakeholders during project start-up and design activities. For clinical staff, the prospect of enhanced care through more frequent patient-clinician communication, remote monitoring, and change triggers was significant. For the service manager and funders, the capacity to optimize resources while retaining care standards was pivotal. For patients and carers, connectedness through continuous monitoring and videoconferencing was attractive. For researchers and technology developers, the chance to demonstrate feasibility and to assess effectiveness was important. This range of views and attitudes informed the concept design and testing specifications as well as the research and evaluation processes.

These processes of negotiation and clarification led the PCTRT to realize that, in order to bring about the desired outcomes, the palliative care service needed to see that the telehealth intervention had a direct and real clinical value for the patient and the carer. This central proposition guided a series of decisions during design and prototype testing.

### Design

**Figure 2:**
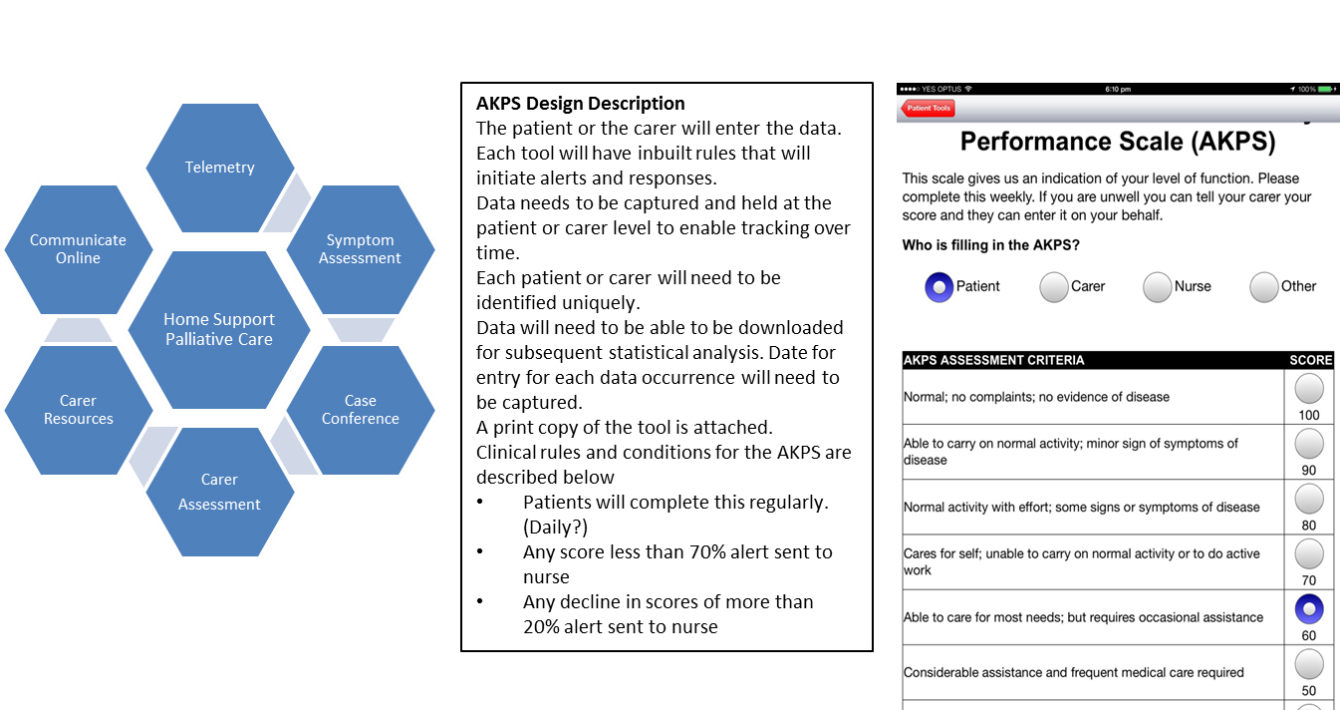
Development from concept ideation to functional clinical description to prototype.

The design process involved careful description of the standard care processes delivered by the palliative care community team and an analysis of how data captured through patient and carer self-report in the community could be integrated into work processes and data systems. This clinical review provided the framework for decision points that needed to be built into the telehealth functionality. While a more detailed technical specification would be developed, this practical specification represented the point of transition between the clinical perception of telehealth as a possibility and the technical production of functional and robust prototypes. Figure 2 outlines the development in the design from initial concept description through clinical articulation to prototype.

While other elements such as the hardware and networks for delivery of the telehealth intervention could be led by the technical team, detailed clinical leadership in defining the characteristics and logistics of the telehealth application during design was fundamental to developing resources that could be acknowledged as valuable in the clinical setting.

## Discussion

### Summary

The focus of this paper is on the complexity of the environment in which decisions that shape the nature and development of a telehealth concept are made. Moving from idea to application is not linear but iterative, informed by what is learned and what is experienced. Feedback, testing, and incorporation of multiple perspectives can enhance the quality and the utility of telehealth modules. Initial concept decisions that reflect a clear and apparent central value proposition but that respect diversity in values among the stakeholders provide a strong basis in moving from idea to practical resource. This process particularly needs to be shaped by critical input from those who will need to engage with the module, most notably consumers (patients and carers) and health professionals [26]. However, even in initial concept discussions, enabling multidisciplinary participation offers an environment where the relative weight of decisions can be tested and determined, providing some assessment of possible return for effort and technical feasibility. Early engagement with those from a range of discipline-relevant backgrounds and with different roles and experiences helps to adjust the attitudes of participants and build an atmosphere that encourages exchange and inclusion.

The importance of the multidisciplinary team was demonstrated throughout the planning and design work as choices about platforms, devices, and systems all had the potential to affect the experience of both patients and clinicians. For example, the clinical understanding of the functional and cognitive capacities of the patient population became an important element in highlighting the relative importance of simplicity of use for a videoconferencing system over advanced functionalities and security settings. Cost and security options for different approaches were also robustly debated given the implications for post-trial sustainability. These discussions again reflected different values held by different participants and stakeholders. For example, systems that could engage with hospital record systems could not be deployed for patient use in the community. Provision of tablets rather than self-contained commercial products were seen to offer the best option for service continuation after the trial at the expense of some functionality that could be used by older people with impairments in physical and cognitive function.

Meetings between the PCTRT and the technical advisors also provided a forum where complex problems could be reviewed from clinical, technical, and research perspectives. For example, in palliative care community outreach, death is seen as an expected event. However, the issue of how to handle the effect of the death of a patient within the telehealth environment requires careful analysis. While the telehealth intervention provides support to both the patient and the carer in their home, the death of the patient would mean there is a need to reassess the carer’s virtual relationship with the service as well as the carer’s experience and use of the tablet in a changed environment, that is, without the presence of their loved one. Various options such as the remote dismantling of the patient resources and/or the enablement of bereavement-specific resources were examined by the PCTRT from the viewpoints of technical feasibility, clinical value, and preferences of the intended user, namely the newly bereaved carer. The resulting dialogue provided the opportunity to illuminate specific aspects of this problem and to examine potential solutions in terms of system capacities and human sensitivities with regard to continuity of relationships and care.

However, while robust discussions about technologies and technical issues were being held at the project level, discussions with the clinical service team identified that many clinicians had no experience and limited confidence in using tablets such as iPads, which were the preferred project device. Early engagement with the clinicians who would be delivering the telehealth service enabled sufficient lead time for clinical members to be provided with training and experience in using the technology that would be provided to patients. This meant that, at the point where clinical staff began a quality assurance exercise with patients, they felt comfortable in introducing patients to the tablet and the apps contained on them.

The CeHRes framework highlights the implicit diversity in views and opinions that can lead to potential divergence in values and competencies among participants and stakeholders. Such inherent conflict requires those involved in planning and developing to be able to articulate underlying assumptions and be involved in the assessment of the relative importance of different options. Greenhalgh et al’s discourse analysis [27] identified conflicting but intersecting discourses on telehealth that reflected different views and values of participants and protagonists in the health sector. Their proposal that learning communities are needed to bridge these gaps reflects the values specification aspect of the CeHRes framework. It also reinforces the need for the initial and ongoing involvement of different parties.

The difficulty is in forming and maintaining teams that can assimilate these varying perspectives and strengthen the developed resources by accommodating technical, clinical, and political complexity. Informed collective decision making assumes that technical, clinical, and social decisions should not be made independently. However, this also implies that participants are able to deal with potentially uncomfortable and unfamiliar sets of knowledge to ensure that these perspectives are integrated. While such team input provides a rich appreciation of the complex nature of the task being undertaken, it also means that there is a need to acknowledge and understand the expertise and roles of different contributors. The challenges associated with understanding non-shared concepts and terminologies and respecting different processes and paradigms has been previously reported [28]. However, it is also important to acknowledge that establishing working relationships within and across the clinical and technical teams requires a substantial contribution of time and focus. This was challenging in this project as all members of the PCTRT, except the project manager, were participating in the project in addition to their normal clinical and management responsibilities. Tensions associated with positional authority, inability to attend all activities that could influence the design and development of the telehealth modules, and the persistent and detailed analysis and specification required, created pressures for participants and for teams. So, given that the time commitment needed to ensure purposeful engagement and contribution is significant, and recognizing that it is this contribution that drives the contextual inquiry and values analysis, projects need to incorporate this resource requirement adequately into planning.

### Conclusions

Telehealth module development is complex and represents a balance between clinical need, consumer benefit, and technical and financial feasibility. A clear health value proposition appears to provide a basis for measurement of different viewpoints and gives clarity to assessing purpose, application, and effect. There may not be a “perfect” answer for any specific telehealth intervention, so the articulation of stages and activities in the CeHRes model that can guide the development and uptake of eHealth provides a mechanism to support “better” solutions that have addressed the underlying, and often unstated, values and beliefs of different participants.

## Abbreviations

CeHRes: Center for eHealth Research
PCTRT: Palliative Care (Telehealth) Research Team
